# Cognitive behavioural therapy interventions for insomnia among shift workers: RCT in an occupational health setting

**DOI:** 10.1007/s00420-019-01504-6

**Published:** 2019-12-18

**Authors:** Heli Järnefelt, Mikko Härmä, Mikael Sallinen, Jussi Virkkala, Teemu Paajanen, Kari-Pekka Martimo, Christer Hublin

**Affiliations:** 1 Finnish Institute of Occupational Health (FIOH), Topeliuksenkatu 41 b, 00250 Helsinki, Finland; 2 Department of Psychology and Logopedics, University of Helsinki, Helsinki, Finland; 3 Department of Psychology, University of Jyväskylä, Jyväskylä, Finland; 4 Department of Clinical Neurophysiology, University of Helsinki and Helsinki University Hospital, Helsinki, Finland

**Keywords:** Insomnia, Cognitive behavioural therapy for insomnia, Self-help, Sleep hygiene education, Shift work, Shift work disorder, Occupational health services

## Abstract

**Introduction:**

The aim of the study was to compare the effectiveness of cognitive behavioural therapy interventions for insomnia (CBT-I) to that of a sleep hygiene intervention in a randomized controlled design among shift workers. We also studied whether the features of shift work disorder (SWD) affected the results.

**Methods:**

A total of 83 shift workers with insomnia disorder were partially randomized into a group-based CBT-I, self-help CBT-I, or sleep hygiene control intervention. The outcomes were assessed before and after the interventions and at 6-month follow-up using questionnaires, a sleep diary, and actigraphy.

**Results:**

Perceived severity of insomnia, sleep-related dysfunctional beliefs, burnout symptoms, restedness, recovery after a shift, and actigraphy-based total sleep time improved after the interventions, but we found no significant differences between the interventions. Mood symptoms improved only among the group-based CBT-I intervention participants. Non-SWD participants had more mental diseases and symptoms, used more sleep-promoting medication, and had pronounced insomnia severity and more dysfunctional beliefs than those with SWD. After the interventions, non-SWD participants showed more prominent improvements than those with SWD.

**Conclusions:**

Our results showed no significant differences between the sleep improvements of the shift workers in the CBT-I interventions and of those in the sleep hygiene control intervention. Alleviation of mood symptoms seemed to be the main added value of the group-based CBT-I intervention compared to the control intervention. The clinical condition of the non-SWD participants was more severe and these participants benefitted more from the interventions than the SWD participants did.

**Trial registration:**

ClinicalTrials.gov, NCT02523079.

## Introduction

Insomnia affects a large proportion of the population (Morin et al. 2015). Approximately 30% of the population have recurring insomnia symptoms and 5–10% have chronic insomnia disorder that includes wake-time symptoms (Partinen and Hublin 2011). In Finland, the prevalence of insomnia-related symptoms among the working-age population has increased from 20 to 30% in the 1970s to as high as 40–45% in the 2000s (Kronholm et al. 2016). Identifying and treating insomnia efficiently is important because of its high prevalence and because it increases the risks of both mental and somatic diseases (Baglioni et al. 2012; Sivertsen et al. 2014; Vgontzas et al. 2013) and reduces work and cognitive ability (Fortier-Brochu et al. 2012; Kucharczyk et al. 2012).

Insomnia is even more common among shift workers (Kerkhof 2018). This is probably related to the temporal misalignment of the homeostatic and circadian processes of sleep associated with irregular shifts (West and Bechtold 2015). Today, approximately one-fifth of the European Union (EU) employees work in shifts (Eurofound 2017), making shift workers a significant group of people whose potential insomnia should be managed using effective interventions.

Cognitive behavioural therapy for insomnia (CBT-I) is effective in a variety of patient populations and settings (Morin et al. 2015). Patient-reported outcomes, such as the Insomnia Severity Index (ISI) (Morin et al. 2011), estimate that 70–80% of patients achieve a response, and improvements in sleep continuity and efficiency have medium to large effect sizes on average (Morin et al. 2015). Based on the European guidelines for the treatment of insomnia, CBT-I is recommended as the first-line treatment for chronic insomnia (Riemann et al. 2017). However, only a few CBT-I effectiveness studies have been conducted among shift workers. Earlier non-randomized studies have shown that CBT-I may also be effective among shift workers (Jarnefelt et al. 2012; Peter et al. 2019). On the other hand, one study found that CBT-I implemented at the workplace was only effective when shift workers were not included in the analyses (Schiller et al. 2018).

Shift workers’ irregular sleep–wake patterns are a challenge in the screening and treatment of insomnia. Firstly, it may be difficult to distinguish between insomnia disorder and shift work disorder (SWD) (American Academy of Sleep Medicine 2014). SWD is a clinical circadian rhythm sleep disorder defined as insomnia and/or excessive sleepiness that lasts at least 3 months and is associated with a prolonged shift work schedule that overlaps habitual sleeping time. Its etiology is primarily attributed to circadian disruption and misalignment due to shift work, and its prevalence rate is estimated to be 10–23% among rotating shift and night workers (Wright et al. 2013). A number of treatment efforts have targeted the core features of shift work and focused on improving circadian adaption and sleep and reducing sleepiness (Wickwire et al. 2017). From an organizational perspective, SWD must be handled within the broader context of fatigue risk management and prevention. In practice, SWD and insomnia disorder may coexist, which may complicate screening of and treatment decisions regarding insomnia among shift workers (Drake et al. 2004). For example, insomnia may initially be associated with the shift work schedule but may progress into insomnia even on days off (Wright et al. 2013). Secondly, following a regular sleep/wake pattern is one central principle of CBT-I (Morin et al. 2015) that shift workers cannot do. This is why a modified version of CBT-I has been applied (Jarnefelt et al. 2012).

Because shift workers with irregular schedules may find it difficult to participate in regularly scheduled face-to-face treatment typically used in the group treatment context, self-help treatments, such as mobile applications, are needed (Wickwire et al. 2017). Although self-help CBT-I treatments are shown to be effective (Cheng and Dizon, 2012), they need further validation (Morin et al. 2015). This is particularly the case for shift workers because relatively few controlled intervention studies have been conducted on health and sleep problems and the self-help perspective, in particular, has not yet been systemically studied among this group (Kecklund and Axelsson 2016).

Good sleep hygiene practices are considered a critical step in any sleep treatment approach among shift workers (Wickwire et al. 2017). Sleep hygiene education refers to behavioural, environmental, and other sleep-related recommendations to help patients improve their sleep (Stepanski and Wyatt 2003). However, though epidemiological and experimental research generally supports an association between individual sleep hygiene recommendations and nocturnal sleep, the direct effects of these recommendations on sleep remain largely untested in the general population (Irish et al. 2015). Compared to CBT-I, there is also little evidence that sleep hygiene education alone is effective in the treatment of insomnia disorder (Morin et al. 2015). The role of sleep hygiene education should be compared to that of CBT-I in the treatment of insomnia among shift workers.

As occupational health services (OHS) cover 84% of the employed workforce in Finland (Lappalainen et al. 2016), it is essential that OHS personnel are able to provide effective insomnia treatments. The aim of our study was to evaluate the effectiveness of group-based CBT-I (gCBT-I) and self-help-based CBT-I (sCBT-I) interventions compared to a sleep hygiene (SH) control intervention in a randomized and controlled design among shift workers with insomnia in an OHS setting. We studied the differences between and the effectiveness of treatment methods on self-appraised severity of insomnia, sleep-related dysfunctional beliefs, subjective and objective insomnia symptoms, sleep, and alertness. Based on the bidirectional relationship between insomnia and medical and psychiatric morbidity and quality of life, recommendations for a standard research assessment of insomnia include measuring waking correlates and the consequences of insomnia (Buysse et al. 2006; Morin et al. 2015). In this study, we assessed the differences between the improvements caused by the different interventions to the waking correlates, including burnout symptoms, depression symptoms, trait of worry, and health-related quality of life. Finally, we evaluated whether SWD features were associated with the results. We hypothesized that participants with SWD features benefit less than participants without these features because the insomnia symptoms of SWD are primarily associated with circadian disruption and misalignment due to shift work, which psychological interventions cannot significantly change.

## Methods

### Recruitment and participants

We recruited volunteer shift workers from the personnel of Helsinki city hospitals, the City of Turku, an airline, a bakery, and a media company. Recruitment was organized by OHS and via the intranet or leaflets at the workplaces. In addition, the Finnish Institute of Occupational Health (FIOH) recruited shift workers through advertisements posted in the commercial press, on their website, and on their Facebook and Twitter accounts. Recruitment began in May 2015 or August 2016, depending on the partner, and ended in May 2017. However, the individual assessment and treatment process began immediately after each participant was recruited.

In OHS, the recruitment and assessment process proceeded as follows: (1) A nurse met the interested subject, provided information on the study, and assessed inclusion/exclusion criteria based on the candidate’s age, insomnia duration, working hours, and work situation; (2) suitable candidates kept a sleep diary for 2 weeks and replied to a modified semi-structured sleep and health questionnaire (Partinen and Gislason 1995) and a questionnaire with shift-specific questions on insomnia and sleepiness (SS-Q) (Vanttola et al. 2018) and the Insomnia Severity Index (ISI) (Morin et al. 2011); (3) the candidate attended an appointment with a physician who decided on inclusion/exclusion based on the clinical interview and examination, sleep diary, and questionnaire results. All the selected participants gave their informed consent. The FIOH assessment process had one exception: a sleep medicine specialist (CH) and clinical psychologist and psychotherapist specialized in insomnia and CBT-I (HJ) made preinclusion or exclusion decisions based on short electronic questionnaires on age, working hours, insomnia symptoms, illnesses, and medications. This exception was made because, unlike in the recruiting OHS centers, the participants’ previous health histories were not available at the FIOH. After this preinclusion, the suitable candidates met a nurse.

The FIOH sleep medicine specialist and psychologist gave all the OH nurses and physicians a two-hour course on the study plan, the clinical assessment of insomniacs, and inclusion and exclusion criteria. The physicians and nurses were able to consult the FIOH specialists when needed during the assessments. We used the following inclusion criteria: (1) age of 20‒60 years; (2) non-organic insomnia (F51.0) (World Health Organization 1995); (3) difficulty initiating [sleep onset latency (SOL)] and/or maintaining sleep [wake after sleep onset (WASO)] for ≥ 30 min and/or use of sleep-promoting medication (SPM) at least three nights per week for at least 3 months (Buysse et al. 2006); (4) motivation to receive non-pharmacological treatment for insomnia; (5) full-time (80–100%) shift work, which was defined as working hours consisting of at least 10% of morning shifts (beginning 07:00 or earlier), evening shifts (ending 22:00 or later) or night shifts (at least 3 hours of a shift falling between 23:00 and 06:00). This cut-off score of 10% was considered a minimum exposure level to shift work. In addition, participants had to work at least two shift types (e.g. morning and night shifts) and (6) be fluent in Finnish, as the intervention materials were in Finnish. We considered non-assessed or untreated clinically significant somatic or mental symptoms or illnesses or other sleep disorders that could explain current insomnia symptoms or interfere with or be worsened by CBT-I as exclusion criteria for the study.

**Fig. 1:**
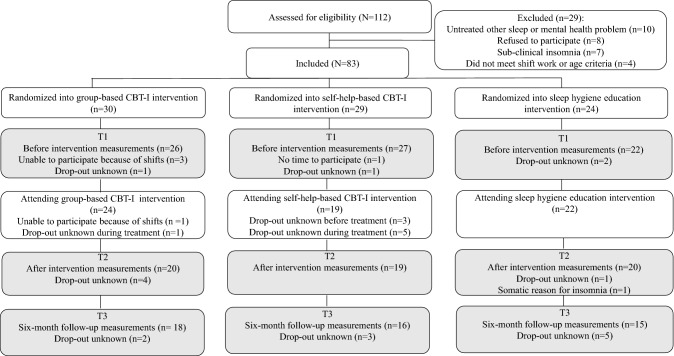
Participant flowchart

Figure 1 represents the participant flowchart. A total of 112 candidates were assessed for eligibility. Other untreated sleep problems, e.g. restless legs syndrome, were the main reason for exclusion (19 women and 10 men; mean (M) age 42.5, standard deviation (SD) 10.4 years). This resulted in a total of 83 participants, who were randomized into the interventions. Eight participants discontinued before the study measurements (6 women, 2 men; M age 42.3, SD 6.2 years).

The demographic and clinical characteristics of the sample attending the before intervention (T1) measurements are presented in Table 1. The participants were middle-aged [median (Mdn) 45, range (RG) 21 − 60 years] and 75% were women. A total of 45% worked in the health and social care e.g. as nurses, 17% worked in a bakery and 12% in the aviation industry e.g. as cabin attendants. One-quarter worked in e.g. the security and land transportation fields. Over half (53%) had a three-shift work schedule, nearly a quarter (24%) worked irregular hours, and the rest had a two-shift work schedule. On average, 16.5 (SD 6.1) of all shifts per month were either early morning (35%; M 5.8, SD 4.9), evening (41%; M 6.7, SD 3.6), or night (24%; M 4.0, SD 3.2) shifts. The total duration of shift work averaged 16 years.

**Table 1:** Demographic and clinical characteristics of participants of different interventions [means (M), standard deviations (SD), medians (Mdn), and ranges (RG)], total sample and separately

Characteristics	Total sample (*N* = 75)	Group-based CBT-I (*n* = 26)	Self-help-based CBT-I (*n* = 27)	Sleep hygiene education (*n* = 22)
Age (years), Mdn (RG)	45 (21 − 60)	45 (22 − 59)	41 (21 − 60)	47 (25 − 59)
Sex, *n* (%)				
Women	56 (75)	18 (69)	21 (78)	17 (77)
Marital status, *n* (%)				
Married/cohabiting	39 (52)	12 (46)	17 (63)	7 (32)
Educational level, *n* (%)				
Lower secondary or primary	26 (35)	11 (42)	10 (37)	5 (23)
Higher secondary	22 (29)	9 (35)	6 (22)	7 (32)
Master’s degree	6 (8)	0	4 (15)	2 (9)
Professional field, *n* (%)				
Health or social care	34 (45)	13 (50)	10 (37)	11 (50)
Bakery industry	13 (17)	7 (27)	4 (15)	2 (9)
Aviation industry	9 (12)	1 (4)	5 (19)	3 (14)
Other	19 (25)	5 (19)	8 (30)	6 (28)
Shift work schedule, *n* (%)				
Three-shift	40 (53)	13 (50)	16 (59)	11 (50)
Irregular	18 (24)	5 (19)	8 (30)	5 (23)
Two-shift excluding night shift	10 (13)	6 (23)	1 (4)	3 (14)
Two-shift including night shift	7 (9)	2 (8)	2 (7)	3 (14)
Duration of shift work (years), M (SD)	16.3 (8.9)	14.3 (0.4)	17.3 (9.3)	17.6 (7.6)
Duration of insomnia, years, Mdn (RG)	5.0 (0.75 − 25)	6.5 (1 − 25)	5.0 (0.83 − 20)	3.0 (0.75 − 20)
ISI during assessment phase, M (SD)^a^	16.3 (4.3)	16.3 (4.2)	16.4 (4.6)	16.3 (4.3)
Sleep-promoting medication, *n* (%)	48 (64)	17 (65)	19 (70)	12 (55)
Features of shift work disorder, *n* (%)	37 (49)	10 (38)	12 (44)	15 (68)
Comorbid diseases, *n* (%)				
None	31 (41)	9 (35)	11 (41)	11 (50)
Physical	38 (51)	14 (54)	13 (48)	11 (50)
Mental	6 (8)	3 (12)	3 (11)	0

^a^Insomnia Severity Index [no insomnia (0 − 7 points), subthreshold insomnia (8 − 14 points), moderate (15 − 21) or severe (22 − 28 points) insomnia]

Participants had suffered from insomnia from 9 months to 25 years. The ISI sum score (Bastien et al. 2001) in the assessment phase showed moderately severe clinical insomnia on average. Sixty-four per cent had used SPM during the last 3 months (31% at least three times a week, 11% once or twice a week, and 23% less often). SWD features were evaluated based on the SS-Q and the sleep diary from the assessment phase. A participant was evaluated as having SWD if they had suffered from insomnia symptoms and/or sleepiness during the last 3 months always or often during the early morning, evening, and/or during the night shifts, and never or rarely on holidays and days off (Vanttola et al. 2018). In accordance to the clinical criteria of SWD (American Academy of Sleep Medicine 2014), based on their sleep diaries the participants also had to have averaged at least one hour less sleep on the early morning, evening, and/or night shift days than on day shift days or days off. Based on these criteria, the number of SWD and non-SWD participants equaled one and other (one participant was excluded from the analysis because her SWD features could not be evaluated, as she had not worked shifts during the assessment phase). Approximately half of the participants (51%) had some diagnosed comorbid physical disease, most commonly hypertension (10 participants), allergy (9), hypothyroidism (9), migraine (7), and pain symptoms (7); and 8% had a diagnosed mental disease, most commonly depression (6). However, all physical and mental diseases were under balanced treatment. β-blockers (10 participants), antihistamines (9), and thyroxine (9) were the concurrent pharmacological treatments for comorbid diseases. The described demographic and clinical characteristics of the participants randomized into the interventions did not differ significantly when we accounted for those who discontinued before T2 measurements or when we excluded them.

The participants recruited by OHS (50 participants) differed from those recruited by FIOH (25 participants) in two demographic characteristics. The participants recruited by OHS had longer experience with shift work than those recruited by FIOH [t_73_ = 3.00, p = 0.004; on average 18.4 (SD 8.6) vs. 12.2 (SD 8.1) years]. They also differed in terms of their professional fields ($\chi_{3}^{2}$ = 19.04, *p* < 0.001). The proportions of bakery (OHS 26% vs. FIOH 0%) and aviation (16% vs. 4%) industry workers were larger in the OHS recruits than in the FIOH recruits. In contrast, the proportion of OHS recruit participants working in other fields (12%) was lower than among those recruited by FIOH (52%). The proportion of participants working in the health and social care field (46% vs. 44%) did not differ between the recruitment procedures.

### Study design

The study design was planned as a clinical randomized controlled trial (RCT). A total of 83 participants were randomized into gCBT-I, sCBT-I, or SH education (Fig. 1). Randomization was conducted using adaptive stratified sampling (minimization) so that age, gender, and the ISI sum score during the assessment phase averaged the same in each intervention type. However, randomization was only partial because aviation industry participants were not randomized into the gCBT-I due to unsuitability of the group treatments in their OHS. The measurements were conducted before intervention (T1), after the intervention (T2), and at a 6-month follow-up (T3). During the T1, T2, and T3 phases 90%, 71%, and 59% of the included participants attended the measurements, respectively.

### Measures

The primary outcome measure was perceived severity of insomnia, measured with the ISI sum score (Bastien et al. 2001).

Insomnia symptoms and sleep were assessed using a sleep diary (Carney et al. 2012), which contained questions on SOL, WASO, time in bed (TIB), and restedness after sleep. Total sleep time (TST) (= TIB – SOL – WASO) and sleep efficiency (SE) (= TST/TIB × 100) were calculated afterwards. Participants additionally appraised their recovery after a shift (Kinnunen and Feldt 2013). They kept a sleep diary for 2 weeks during all the measurement phases.

We also evaluated insomnia symptoms and sleep using GENEActive (Activinsights Ltd. Cambs, UK) actigraphs. Actigraph recordings were conducted while the sleep diaries were being kept. The participants wore the actigraphs on their non-dominant wrists for 24 h a day and were instructed to press a button when they began trying to sleep and when they woke up. The epoch length used was 1 min. From the actigraph data, we analysed four sleep parameters (SOL, WASO, TST, and SE) using Sleep Analysis 7.40 (CamNtech, Cambridge, UK) and medium sensitivity.

In addition to the ISI, participants completed other questionnaires at each measurement phase.

Sleep-related dysfunctional cognitions were assessed using the 16-item version of Dysfunctional Beliefs and Attitudes about Sleep (DBAS) (Morin et al. 2007). Symptoms of burnout were assessed using the Shirom-Melamed Burnout Measure (SMBM) (Shirom and Melamed 2006). Depression symptoms were assessed using the Beck Depression Inventory (BDI) (Beck et al. 1988). Trait of worry was assessed using the Penn State Worry Questionnaire (PSWQ) (Meyer et al. 1990). Health-related quality of life was assessed using the Finnish version of the RAND 36-Item Health Survey, which measures eight facets of the quality of life (Aalto et al. 1999; Hayset al. 1993). Two summary scores used in the previous study (Jarvinen et al. 2003) served as the final scores: the physical component summary (PCS) and the mental component summary (MCS). In addition, the participants evaluated treatment effectiveness after the intervention and at a 6-month follow-up by answering a question from the Consumer Reports Survey (CRS) (Seligman 1995) on the treatments’ global effectiveness in improving their overall life situations and well-being. The participants filled out the questionnaires using the Internet-based programmes Digium (until July 2017) and Questback (after July 2017), to which they had their own Secure Sockets Layered (SSL) link.

### Interventions

The *gCBT*-*I* intervention consisted of six group sessions (each lasting 90 min) led by a psychologist. The first five sessions were held weekly and the last one 4 weeks after the fifth session. Each group consisted of four to six participants, who received a workbook containing instructions and tasks for each session (60 pages in total).

The *sCBT*-*I* intervention consisted of six recorded slide show sessions (each lasting 10 − 35 min) and four relaxation and mindfulness recordings (each lasting 8 − 12 min) that the participants watched and listened to on a tablet computer they received for the treatment period. Each slide show and additional recordings were scheduled to be activated weekly on the computer. Participants additionally received the same workbook as the gCBT-I intervention participants. Participants had one individual session with a nurse before the treatment, during which the nurse gave them instructions on how to use the self-help material. The session after the self-help programme was used to review each participant’s feedback and experiences (both lasting 30 min).

The *SH* education intervention consisted of one individual session (45 min) delivered by a nurse. She told the participant about sleep hygiene and gave them a short workbook containing instructions (six pages) and space for the participant’s personal goals to improve their sleep hygiene.

The contents of the treatments are summarized in Table 2. The CBT-I interventions were based on the general CBT-I model (Morin et al. 2015). However, we modified the behavioural methods and sleep and alertness hygiene instructions, as in our earlier study (Jarnefelt et al. 2012). Behavioural sleep restriction was modified so that one extra hour in bed was allowed in one to three sleep periods in cases of substantial shift work-related sleep debt. In addition, participants received information on how to schedule sleep, manage sleep debt, or promote sleep and alertness while working in shifts: e.g. well-timed naps, eating and exercise, and the use of eye covers and earplugs while sleeping. The contents of the SH education were similar to the sleep and alertness hygiene contents (Session two) of the CBT-I interventions.

**Table 2:** Summary–contents of group and self-help-based sessions of cognitive behavioural therapy for shift workers with chronic insomnia. The contents of the sleep hygiene education were similar to that of session two, excluding relaxation training

**Session 1: sleep information**
Normal sleep processes (e.g. sleep physiology), insomnia, sleep and wakefulness during different shifts, and treatment principles
Individual factors affecting sleep and insomnia
Individual goals for the treatment
Abbreviated progressive relaxation training
**Session 2: sleep and alertness hygiene**
Sleep hygiene instructions (e.g. caffeine consumption and pre-sleep routines)
Alertness hygiene instructions (e.g. well-timed naps and other breaks)
Making plans for improving sleep and alertness hygiene
Cue-controlled relaxation training
**Session 3: behavioural methods and hypnotic discontinuation**
Scheduling sleep, wake, and light in different shifts based on circadian principles
Reshaping sleep patterns (stimulus control or modified sleep restriction)
Individual plan for hypnotic discontinuation if needed
**Session 4 and 5: cognitive methods**
Sleep-disturbing cognitive processes
Developing alternative ways to handle planning, worrying, and thought racing before bedtime and at night (cognitive worry control, thought blocking, peaceful place imagery training, and mindfulness training)
Cognitive restructuring of individual sleep-disturbing beliefs and attitudes to sleep and insomnia
**Session 6: follow-up, refresher of CBT principles, and feedback**
Follow-up through sleep diary
Integrating and seeking advice from previous sessions. Compiling an individual summary of helpful methods and how to maintain implementation after treatment
Instructions for possible recurrence of insomnia symptoms
Evaluating the helpfulness of treatment

The five gCBT-I groups had a total of 24 participants (Fig. 1). Eleven participants (42%) attended five to six sessions (only one attended all six sessions), nine participants (35%) attended three to four sessions, and four participants (19%) attended one to three sessions. If they missed sessions, the participants “caught up” via a workbook and short discussions in the next session. The sCBT-I sessions had 24 participants altogether, five of which only attended the first introduction session and discontinued the study before the feedback session. The SH intervention session had 22 participants.

### Treatment providers

The gCBT-I sessions were led by four psychologists and the sCBT-I and SH sessions by 16 nurses from the OHS units. We used the following methods to ensure the integrity of treatment allocations: (1) Nurses and psychologists participated in a short course on insomnia, CBT-I principles, and instructions for the interventions. The course comprised 12 h, split into two training sessions led by the FIOH psychologist; (2) The CBT-I and SH materials modified for a shift work setting were created by the FIOH psychologist and the sleep medicine specialist. The materials comprised take-home workbooks for the participants, PowerPoint presentations for each session, and a guide for treatment providers containing implementation guidelines; (3) the FIOH psychologist offered the OH nurses and psychologist an ongoing mentoring option.

### Statistical methods

Power calculations conducted during the planning stage of the study were based on the average clinically significant improvement in ISI (Morin et al. 2011), the SDs in the ISI, and the number of dropouts in our previous CBT-I study (Jarnefelt et al. 2012). The calculations revealed that a total sample size of 90 individuals (30 per group) had 90% power (α = 0.05, two tail) to reject the null hypothesis of no significant improvement in insomnia severity following the CBT-I treatments.

We assessed the possible differences in the demographic or clinical characteristics between participants randomized into different interventions using One-Way ANOVAs if a variable was normally distributed and using the Kruskal–Wallis or *χ*^2^ tests if a variable was not normally distributed. Likewise, we assessed the possible differences between the demographic and clinical characteristics of the participants recruited by the OHS centers and FIOH, and in addition, the differences in the outcomes over the measurement phases between the SWD and non-SWD participants using independent *t* tests, Mann–Whitney *U* tests, or *χ*^2^ tests.

We analysed the results of the participants who had participated in both the T1 and T2 measurements. We used an intention-to-treat analysis at T3, in which each missing value was replaced by the value from the T2 phase, respectively, using the methodology of an earlier trial of insomnia (Jacobs et al. 2004).

For further analyses, the participants were divided into three response groups based on the magnitude of ISI reduction at T3: (1) The *Responders’* ISI had decreased by at least seven points, representing at least a one-category reduction in ISI (Bastien et al. 2001); (2) the Partial responders’ ISI had decreased by three to six points, representing only a minor change in insomnia symptoms; and (3) the Non-responders’ ISI had not decreased by more than two points, had not changed at all, or had increased all indicating no improvement in symptoms.

We analysed the main effects and the interactions in each outcome variable over the measurement phases and with contrasts (T1-T2 and T1-T3) to compare both CBT-I interventions to the SH control intervention and to compare SWD and non-SWD participants using a general linear model (ANOVA with repeated measurements) if the variable was normally distributed. We used a generalized linear model (using Wald *χ*^2^) with an exchangeable working correlation matrix if the variable was not normally distributed. The *χ*^2^ -test was used to compare both CBT-I interventions to the SH intervention and to compare SWD and non-SWD participants in the deviations of the three treatment response groups. A *p* value of < 0.05 was considered statistically significant. We calculated the effect sizes (Cohen’s *d*) of the statistically significant results to estimate impact.

We used IBM SPSS Statistics version 25 for the analyses.

## Results

### Questionnaire results

Neither the gCBT-I nor the sCBT-I intervention differed from the SH control intervention in terms of the reduction of the main outcome of the study, i.e. the perceived severity of insomnia (ISI). Regardless of the intervention, ISI decreased (23% from T1 average) significantly over the measurement phases (*F*_2,112_ = 21.04, p < 0.001). Compared to the T1 phase, ISI decreased during the T2 phase (*F*_1,56_ = 33.85, *p* < 0.001, *d* = 0.68) and at T3 (*F*_1,56_ = 25.58, *p* < 0.001, *d* = 0.63).

Based on the above-mentioned ISI response grouping, the interventions differed in the reduction magnitude of ISI ($\chi_{4}^{2}$ = 8.62, *p* = 0.07), showing a trend that more respondents were in the gCBI-I (40%) than in the SH control intervention (5%). The number of respondents in the sCBT (16%) and SH intervention did not differ significantly. Respectively, there were more partial respondents in the SH (45%) than in the gCBT-I intervention (20%). The number of partial respondents did not differ significantly between the sCBT (32%) and SH control intervention. The number of non-respondents did not differ significantly (gCBT-I 40%, sCBT-I 53%, and SH 50%).

We found no significant differences in the reduction of sleep-related dysfunctional beliefs measured by DBAS between the CBT-I and SH control intervention participants. Regardless of the intervention, beliefs decreased significantly (11%) over the measurement phases (*F*_2,112_ = 12.77, *p* < 0.001). Compared to the T1 phase, DBAS decreased during the T2 phase (*F*_1,56_ = 15.32, *p* < 0.001, *d* = 0.38) and at T3 (*F*_1,56_ = 27.70, *p* < 0.001, *d* = 0.38).

Neither the gCBT-I nor the sCBT-I intervention differed from the SH control intervention in terms of the reduction of burnout and stress symptoms measured by SMBM. Regardless of the intervention, SMBM decreased significantly (11%) over the measurement phases (*F*_2,112_ = 9.28, *p *< 0.001). Compared to the T1 phase, SMBM decreased during the T2 phase (*F*_1,56_ = 13.69, *p* < 0.001, *d* = 0.40) and at T3 (*F*_1,56_ = 13.25, *p* = 0.001, *d *= 0.40).

The interaction effect of depression symptoms measured by BDI between the interventions was non-significant, but we observed a trend in which symptoms decreased significantly more at T3 among the gCBT-I intervention participants than among those in the SH control intervention ($\chi_{1}^{2}$ = 4.04, *p* = 0.044, *d* = 0.54). Regardless of the intervention, depression symptoms decreased (43%) significantly during the measurement phases ($\chi_{2}^{2}$ = 8.75, *p* = 0.013). Compared to the T1 phase, BDI had decreased at T3 ($\chi_{1}^{2}$ = 8.48, *p* = 0.004, *d* = 0.82).

An interaction effect occurred in the trait of worry measured by PSWQ ($\chi_{2}^{2}$ = 7.01, *p* = 0.030), showing a greater decrease at T3 among the gCBT-I intervention participants than among the SH control intervention participants ($\chi_{1}^{2}$ = 3.83, *p* = 0.050, *d *= 0.53). Regardless of the intervention, *trait of worry* decreased significantly (10%) over the measurement phases ($\chi_{2}^{2}$ = 16.67, *p* < 0.001). Compared to the prior intervention phase, PSWQ had decreased at T3 ($\chi_{1}^{2}$ = 15.69, *p* < 0.001, *d* = 1.20).

The physical component of the health-related quality of life measured by RAND showed no significant differences between the interventions or showed significant changes regardless of the intervention. We also found no significant differences in the improvement of the mental component of the quality of life among both the CBT-I and SH control intervention participants. Regardless of the interventions, we observed a trend in which the mental quality of life improved (9%) over the measurement phases ($\chi_{2}^{2}$ = 5.84, *p* = 0.054). Compared to the T1 phase, the mental quality of life had improved at T3 ($\chi_{1}^{2}$ = 5.23, *p* = 0.022, *d* = 0.62).

The participants’ evaluations of the global effectiveness of the interventions in improving their overall life situations and well-being differed ($\chi_{2}^{2}$ = 9.32, *p* = 0.009). Paired comparisons showed that the sCBT-I intervention participants ($\chi_{1}^{2}$ = 4.40, *p* = 0.036, *d* = 0.57) and indicatively also the gCBT-I intervention participants ($\chi_{1}^{2}$ = 3.35, *p* = 0.067, *d* = 0.49) evaluated greater improvement than the SH control intervention participants. The evaluations did not change significantly at T3.

The summary data of all the questionnaire results are represented in Table 3.

**Table 3:** Means (M) and standard deviations (SD) or medians (Mdn) and ranges (RG) of questionnaire outcomes at the before intervention (T1), after intervention (T2), and six-month follow-up (T3) phases in the total sample and separately among the participants of the different interventions

Questionnaires	Total sample (*n* = 59)	Group-based CBT-I (*n* = 20)	Self-help-based CBT-I (*n* = 19)	Sleep hygiene education (*n* = 20)
ISI, M (SD)^a^				
T1	14.0 (5.0)	15.4 (5.0)	13.6 (5.2)	13.0 (4.8)
T2	10.7. (4.7)	10.9 (5.4)	10.7 (4.4)	10.6 (4.7)
T3	10.8 (5.1)	11.1 (5.7)	10.7 (4.9)	10.6 (5.0)
DBAS, M (SD)^b^				
T1	5.4 (1.6)	5.5 (1.5)	6.0 (1.3)	4.8 (1.7)
T2	4.8 (1.6)	4.8 (1.4)	5.0 (1.8)	4.6 (1.7)
T3	4.8 (1.6)	4.7 (1.8)	5.2 (1.4)	4.4 (1.7)
SMBM, M (SD)^c^				
T1	3.7 (1.0)	3.9 (1.2)	3.8 (0.7)	3.5 (0.9)
T2	3.3 (1.0)	3.3 (1.1)	3.1 (0.9)	3.3 (0.9)
T3	3.3 (1.0)	3.4 (1.1)	3.5 (0.7)	3.2 (1.0)
BDI^d^, Mdn (RG)^d^				
T1	7 (0 − 24)	9 (0 − 24)	7 (1 − 15)	6.5 (0 − 18)
T2	5 (0 − 21)	7 (0 − 20)	4 (0 − 17)	5 (0 − 21)
T3	4 (0 − 23)	4 (0 − 14)	3 (0 − 17)	5 (0 − 23)
PSWQ, Mdn (RG)^e^				
T1	40 (23 − 66)	47.5 (23 − 66)	40 (24 − 60)	38.5 (26 − 63)
T2	35 (18 − 66)	42.5 (18 − 66)	34 (21 − 64)	32 (23 − 64)
T3	36 (16 − 65)	37 (16 − 63)	38 (24 − 60)	31 (22 − 65)
RAND-PCS, Mdn (RG)^f^				
T1	81.3 (40.0 − 100)	75.6 (47.5 − 100)	83.8 (50.6 − 100)	81.6 (40.0 − 97.5)
T2	85.6 (33.6 − 100)	79.7 (38.8 − 100)	86.3 (45.0 − 97.5)	82.2 (33.8 − 97.5)
T3	85.0 (30.6 −98.8)	89.1 (30.6 − 98.8)	81.9 (50.0 − 96.3)	85.3 (40.6 − 97.5)
RAND-MCS, Mdn (RG)^f^				
T1	73.9 (19.6 − 94.3)	70.8 (19.6 − 94.3)	79.6 (42.3 − 92.8)	74.9 (40.8 − 91.0)
T2	76.0 (37.7 − 96.5)	73.8 (37.7 − 93.3)	83.1 (59.9 − 96.5)	77.8 (49.3 − 88.0)
T3	80.8 (37.7 − 100)	75.4 (37.7 − 100)	84.8 (52.7 − 92.8)	81.1 (44.2 − 96.5)
CRS, Mdn (RG)^g^				
T2	4 (1 − 6)	5 (1 − 6)	5 (1 − 6)	2 (1 − 5)
T3	3.5 (1 − 6)	4 (1 − 6)	4 (1 − 6)	2 (1 − 4)

^a^Insomnia Severity Index (scale 0 − 28)

^b^Dysfunctional beliefs and attitudes about sleep (0 − 10)

^c^Shirom-Melamed burnout measure (1 − 7)

^d^Beck depression inventory (0 − 63)

^e^Penn State Worry Questionnaire (16 − 80)

^f^RAND SF-36 (0 − 100): physical component summary (PCS), mental component summary (MCS)

^g^Consumer Reports Survey question concerning the global effectiveness of treatments to improve overall life situations and well-being [1(not at all)–7(decisive impact)]

### Sleep diary

SOL, WASO, SE, and the usage of SPMs did not differ between the interventions or show significant changes regardless of the intervention. The ranges of SOL, WASO, and SE were quite large, but these outcomes were not generally significantly impaired even during the T1 phase (Mdns of SOL 23 min, WASO 20 min, and SE 90%). Likewise, the usage of SPMs varied from 0 to 100% for all nights (Mdn 7.1%).

TST showed an interaction effect between interventions (*F*_4_ = 2.57, *p* = 0.043). TST showed an increasing trend (12 min) during the T2 phase among the participants of both CBT-I-interventions but decreased, respectively, among the SH education participants (*F*_2_ = 2.46, *p* = 0.095).

We found no significant response differences either in self-reported restedness after a sleep period or in recovery after a shift between the CBT-I and SH control intervention participants. Both outcomes improved significantly (both 7%) regardless of the intervention (*F*_2,100_ = 4.35, *p* = 0.006; *F*_2,102_ = 5.35, *p* = 0.006). Compared to the T1 phase, restedness and recovery experience had increased at T3 (*F*_1,51_ = 6.63, *p* = 0.013, *d* = 0.40; *F*_1,50_ = 7.41, *p* = 0.009, *d* = 0.27, respectively).

Table 4 represents the summary data of the sleep diary results at all measurement phases.

**Table 4:** Medians (Mdn) and ranges (RG) or means (M) and standard deviations (SD) of sleep diary outcomes at the before intervention (T1), after intervention (T2), and 6-month follow-up (T3) phases in the total sample and separately among the participants of the different interventions

Sleep Diary	Total sample (*n* = 54)	Group-based CBT-I (*n* = 20)	Self-help-based CBT-I (*n* = 17)	Sleep hygiene education (*n* = 17)
SOL (min), Mdn (RG)^a^				
T1	22.7 (4.3 − 74.6)	20.0 (4.3 − 74.6)	20.0 (7.9 − 57.5)	28.9 (6.9 − 60.7)
T2	21.9 (3.6 − 73.9)	22.0 (5.0 − 64.6)	23.4 (3.6 − 73.9)	19.4 (6.6 − 73.2)
T3	20.3 (3.9 − 93.6)	18.4 (3.9 − 64.6)	22.0 (6.8 − 76.4)	20.7 (4.9 − 93.6)
WASO (min), Mdn (RG)^b^				
T1	19.6 (0 − 147.1)	20.4 (0.1 − 147.1)	15.0 (1.6 − 55.4)	23.6 (0 − 98.1)
T2	22.9 (0 − 103.8)	24.8 (0 − 84.8)	22.3 (0 − 63.9)	23.6 (0 − 103.8)
T3	21.4 (0 − 126.1)	35.4 (0 − 126.1)	22.5 (1.1 − 49.3)	14.7 (0 − 99.5)
TST (h), M (SD)^c^				
T1	7.0 (0.9)	6.7 (0.8)	7.2 (0.8)	7.2 (1.1)
T2	7.1 (1.0)	6.9 (0.7)	7.4 (1.0)	7.0 (1.3)
T3	7.2 (1.0)	7.1 (0.8)	7.1 (0.9)	7.4 (1.2)
SE (%), Mdn (RG)^d^				
T1	89.8 (66.6 − 97.8)	90.5 (66.6 − 96.0)	90.0 (78.5 − 96.9)	87.5 (76.2 − 97.8)
T2	89.4 (69.6 − 98.5)	90.3 (73.8 − 96.6)	91.7 (79.8 − 98.5)	88.6 (69.6 − 98.5)
T3	89.5 (73.1 − 98.3)	88.7 (73.1 − 96.0)	89.2 (76.7 − 96.0)	91.3 (79.0 − 98.3)
SPM, Mdn (RG)^e^				
T1	7.1 (0 − 100)	15.7 (0 − 100)	21.4 (0 − 100)	0 (0 − 100)
T2	7.1 (0 − 100)	0 (0 − 100)	7.1 (0 − 100)	0 (0 − 100)
T3	0 (0 − 100)	0 (0 − 100)	7.1 (0 − 100)	0 (0 − 100)
Restedness, M (SD)^f^				
T1	2.8 (0.5)	2.9 (0.4)	2.7 (0.5)	2.9 (0.6)
T2	2.7 (0.5)	2.7 (0.5)	2.6 (0.5)	2.8 (0.6)
T3	2.6 (0.5)	2.7 (0.5)	2.6 (0.5)	2.6 (0.4)
Recovery, M (SD)^g^				
T1	2.7 (0.7)	2.7 (0.6)	2.6 (0.6)	2.7 (0.8)
T2	2.7 (0.7)	2.8 (0.6)	2.6 (0.8)	2.8 (0.6)
T3	2.5 (0.8)	2.6 (0.8)	2.3 (0.9)	2.6 (0.7)

^a^Sleep-onset latency

^b^Wake after sleep onset

^c^Total sleep time

^d^Sleep effiency

^e^Sleep-promoting medication (% of nights)

^f^Restedness after sleep period 1(good)−5(poor)

^g^Recovery after a shift 1 (well)–5(poorly)

### Actigraphy

SOL, WASO, and SE did not differ between the interventions or show significant changes regardless of intervention. Similar to the sleep diary results, the ranges of the actigraphy outcomes were quite large, but SOL and SE were not generally significantly impaired during the T1 phase (Mdns of SOL 7 min and SE 86%). However, actigraphy-based WASO was long during the T1 phase (Mdn of WASO 48 min).

We also found no significant differences in the improvement of TST when we compared the CBT-I to the SH control intervention participants. Regardless of the intervention type, TST improved significantly (F_2,98_ = 3.42, p = 0.037). Compared to T2, TST had increased (12 min) at T3 (F_1,49_ = 5.42, p = 0.024, d = 0.24).

The summary data of the actigraphy results in all measurements phases are presented in Table 5.

**Table 5:** Medians (Mdn) and ranges (RG) or means (M) and standard deviations (SD) of actigraphy outcomes at the before intervention (T1), after intervention (T2), and six-month follow-up (T3) phases in the total sample and separately among participants of different interventions

Actigraphy outcomes	Total sample (*n* = 52)	Group-based CBT-I (*n* = 19)	Self-help-based CBT-I (*n* = 18)	Sleep hygiene education (*n* = 15)
SOL (min), Mdn (RG)^a^				
T1	6.9 (0.7 − 23.1)	5.7 (1.4 − 19.9)	6.4 (0.7 − 20.0)	9.9 (1.7 − 23.1)
T2	8.2 (0.4 − 40.2)	6.1 (1.6 − 26.8)	9.3 (2.4 − 40.2)	9.5 (0.4 − 36.4)
T3	8.0 (0.9 − 36.4)	4.0 (0.9 − 29.7)	8.3 (2.4 − 27.4)	8.2 (1.4 − 36.4)
WASO (min), Mdn (RG)^b^				
T1	48.1 (28.7 − 81.7)	43.2 (28.7 − 75.5)	50.4 (30.9 − 81.7)	48.0 (32.1 − 70.0)
T2	45.8 (23.9 − 82.0)	37.1 (26.8 − 82.0)	56.2 (26.6 − 82.0)	44.8 (23.9 − 59.4)
T3	48.2 (23.9 − 86.0)	43.8 (28.0 − 82.7)	53.5 (29.2 − 86.0)	47.4 (23.9 − 70.4)
TST (h), M (SD)^c^				
T1	6.8 (0.7)	6.7 (0.6)	6.7 (0.7)	7.0 (0.8)
T2	6.7 (0.9)	6.6 (0.7)	6.8 (1.0)	6.6 (1.0)
T3	6.9 (0.8)	6.9 (0.7)	6.8 (0.8)	7.0 (1.0)
SE (%), Mdn (RG)^d^				
T1	86.1 (72.2 − 92.6)	88.0 (79.3 − 92.6)	85.5 (72.2 − 90.1)	86.5 (81.2 − 90.5)
T2	86.3 (69.3 − 92.6)	88.1 (75.8 − 92.5)	84.0 (69.3 − 92.0)	87.7 (78.1 − 92.6)
T3	86.0 (72.1 − 92.2)	88.1 (78.4 − 92.2)	85.2 (72.1 − 90.1)	86.0 (78.1 − 91.3)

^a^Sleep-onset latency

^b^Wake after sleep onset

^c^Total sleep time

^d^Sleep effiency

### Further analysis of SWD features

When analysing the demographic and clinical characteristics of the participants with and without SWD features, we found that non-SWD participants had significantly more diagnosed comorbid diseases, especially more comorbid mental disorders ($\chi_{3}^{2}$ = 10.58, *p *= 0.005). All six participants with mental disorders had no SWD, whereas 68% of participants without any comorbid diseases had SWD. In addition, non-SWD participants more commonly used SPM every night or almost every night ($\chi_{4}^{2}$ = 10.41, *p* = 0.034). Fourteen participants used SPM every night or almost every night and 12 of these had no SWD. Non-SWD participants also had a higher ISI during the assessment phase than SWD participants [*t*_72_ = 2.40, *p* = 0.019; ISI: M 17.5 (SD 4.5) vs. M 15.2 (SD 3.9)]. SWD groups did not differ in terms of other demographic or clinical characteristics.

We observed no interaction effect between the SWD groups in terms of the average ISI sum score over the measurement phases or in the ISI-based treatment response group. In other words, both SWD groups improved similarly. However, the average ISI over all the measurement phases was higher among non-SWD participants than among SWD participants [*F*_1_ = 5.55, *p* = 0.022; ISI: M 13.04 (SD 5.7) vs. M 10.5 (SD 4.2)].

An interaction effect of the average DBAS was also non-significant, but a trend emerged showing that at T3 (*F*_1_ = 6.14, *p* = 0.016) DBAS had decreased significantly more among non-SWD participants (reduction of M 1.0, SD 1.1 points) than among SWD participants (reduction of M 0.3, SD 0.8 points). In addition, the average DBAS over all the measurement phases was higher among the non-SWD than among the SWD participants [*F*_1_ = 6.23, *p* = 0.016; DBAS: M 5.4 (SD 1.7) vs. M 4.5 (SD 1.4)].

We found no interaction effect between the SWD groups in terms of BDI. However, the BDI over all the measurement phases was higher among the non-SWD than among the SWD participants [$\chi_{1}^{2}$ = 4.00, *p* = 0.046; Mdn 7 (RG 0 − 24) vs. Mdn 4 (RG 0 − 23)].

We observed an interaction effect between measurement phases and SWD groups ($\chi_{2}^{2}$ = 6.25, *p* = 0.012), showing that at T3, non-SWD participants evaluated the global effectiveness of the intervention better in improving their overall life situations and well-being than SWD participants [$\chi_{1}^{2}$ = 6.25, *p* = 0.012, d = 0.90; Mdn 4 (RG 1 − 6) vs. Mdn 3 (RG 1 − 6)]. We saw no differences between SWD and non-SWD participants in their evaluations of treatment effectiveness over all the measurement phases.

The use of SPM decreased among non-SWD participants (from 44.2 to 30.3% of nights at T2 and to 34.0% of nights at T3) and increased among SWD participants (from 9.7 to 15.0% of nights at T2 and to 10.5% of nights at T3) ($\chi_{1}^{2}$ = 6.15, *p* = 0.013, d = 0.89). The use of SPM was higher among SWD than among non-SWD participants over all the measurement phases [$\chi_{1}^{2}$ = 8.51, *p *= 0.004; Mdn 14.3% of nights (RG 0-100) vs. Mdn 0% of nights (RG 0 − 100)].

The interaction effect of the actigraphic TST was non-significant, but a trend emerged at T3 showing increased actigraphic TST among non-SWD participants (from 6.7 to 7.0 h) and decreased actigraphic TST among SWD participants (from 6.8 to 6.7 h) (*F*_1_ = 8.06, *p* = 0.007). The average actigraphic TST of the SWD and non-SWD participants did not differ over all the measurement phases.

The SWD groups showed no significant differences in term of the other outcomes.

## Discussion

This partially randomized study evaluated the effectiveness of CBT-I interventions among employees whose sleep and wake processes were affected by irregular and sleep-disturbing shifts. Our results showed no significant differences between shift workers’ sleep improvements in the g- and sCBT-I interventions and the SH control intervention. Indicated by a decrease in the perceived severity of insomnia (ISI), insomnia improved moderately among the shift workers after all the interventions. The alleviation of mood symptoms seemed to be the main benefit of gCBT-I intervention compared to the SH control intervention. Half of the participants were evaluated as having features of SWD. In this study, non-SWD participants had a more severe clinical overall picture and benefitted more from the interventions than SWD participants.

The first aim of our study was to evaluate the effectiveness of the g- and sCBT-I interventions among shift workers compared to short SH education. We found no significant differences between the CBT-I interventions and the SH control intervention, nor in the main outcomes of ISI, DBAS, burnout and stress symptoms, or quality of life. These results are in line with a study by Schiller et al. (2018), which showed a significant effect on insomnia symptoms after CBT-I compared to a waiting list control only after shift workers were excluded from the data. However, our previous non-randomized study showed no difference between the improvements among day and shift media workers after CBT-I (Jarnefelt et al. 2014). In addition, a non-randomized study by Peter et al. (2019) showed improvement in ISI among shift workers after online CBT-I. Regardless of the intervention, ISI improved moderately in our current study, and sleep-related dysfunctional beliefs along with burnout and stress symptoms showed small improvements lasting up to the 6-month follow-up after all three interventions. A trend also emerged in which the mental component of health-related quality of life improved moderately at 6-month follow-up. These overall results are parallel to those observed in Peter et al. (2019) but weaker than results obtained in earlier face-to-face and self-help CBT-I studies (Cheng and Dizon 2012; Morin et al. 2015; Okajima et al. 2011) or results seen in our above-mentioned gCBT-I study among shift workers, which showed medium to large effects on ISI, DBAS, and the quality of life after the intervention (Jarnefelt et al. 2012). Based on ISI, 70 − 80% of participants are generally estimated to achieve a response after CBT-I (Morin et al. 1999), while, depending on the intervention, only 5 − 40% of the participants in our current study achieved a response, showing a trend of a better response after gCBT-I than after SH education. This probably reflects that certain shift workers with insomnia benefit more from gCBT than solely from SH education.

The main difference between the studied interventions appeared in the outcomes related to mental health. The gCBT-I of our study was effective in improving the mental health of shift workers, as also observed in earlier studies (Jarnefelt et al. 2012; Okajima et al. 2011; Peter et al. 2019). The trait of worry had decreased substantially at 6-month follow-up, the interaction effect showing a larger reduction after the gCBT-I than SH control intervention. Likewise, depression symptoms decreased at 6-month follow-up and we noted a trend emerging where a significant reduction appeared after gCBT-I. Participants of both CBT-I interventions additionally preferred their interventions’ global effectiveness in improving their overall life situations and well-being compared to the SH education intervention participants. Many explanations potentially exist for the positive mental health effects after CBT-I. On the one hand, as CBT-I partly includes the same components as CBT interventions for mood disorders, it probably directly affects the mood symptoms of insomniacs. On the other hand, better sleep may have indirect positive effects on mental health.

The sleep diary and actigraphy measurements of this study showed no significant changes in SOL, WASO, or SE, which typically improve moderately or substantially after CBT-I (Morin et al. 2015; Okajima et al. 2011). SOL and SE also showed small improvements in our previous study on shift workers (Jarnefelt et al. 2012), and SE improved after both online and face-to-face CBT-I in another study among shift workers (Peter et al. 2019). However, these sleep outcomes were not generally significantly impaired in our current study even before the interventions, except actigraphy-based WASO. The sleep diary-based TST showed a small increase after both CBT-I interventions but a decrease after SH education and actigraphy-based TST showed a small improvement at 6-month follow-up regardless of intervention. Other CBT-I studies have also shown this lagging effect in TST (Okajima et al. 2011). The reason behind this remains unclear but suggests that CBT-I is superior to pharmacotherapy, as effectiveness was sustained and even gradually increased after discontinuation of the treatment. In addition, self-appraised restedness after a sleep period and recovery after a shift showed small improvements at 6-month follow-up in all intervention types. Similarly, restedness improved moderately in the previous study of gCBT-I among shift workers (Jarnefelt et al. 2012). The better restedness and recovery results of our current study may at least partly be due to increased TST along with the better alertness hygiene and relaxation methods used by the participants after the interventions.

The second aim of our study was to evaluate whether SWD features associated with participants’ demographic or clinical characteristics, the study outcomes, and the interventions’ effectiveness. Based on SS-Q (Vanttola et al. 2018) and the sleep diaries, in resemblance to the clinical criteria of SWD (American Academy of Sleep Medicine 2014), half of the participants were evaluated as having SWD features. Compared to them, the clinical picture of non-SWD participants was more severe. Firstly, non-SWD participants had more comorbid diseases, only they had comorbid diagnosed mental disorders, and they used SPMs more frequently over all the measurement phases. Secondly, at the assessment and before the intervention phases, the non-SWD participants perceived their insomnia as more severe, and during the before intervention phase they had more severe sleep-related dysfunctional beliefs and depression symptoms.

We also observed differences in treatment effectiveness depending on the SWD type. Compared to the before intervention phase, SPM usage decreased among the non-SWD participants and increased slightly among those with SWD during both the intervention phase and at 6-month follow-up. We noted two emerging trends at 6-month follow-up: dysfunctional beliefs decreased more among non-SWD participants and actigraphy-based TST increased among non-SWD and decreased among SWD participants. In addition, during the follow-up phase, the non-SWD participants evaluated the global effectiveness of their intervention better. Our results indicate that the main target of non-pharmacological insomnia interventions should be shift workers with insomnia who do not have SWD because their symptoms are more severe. As hypothesized, they also benefited from interventions more than those with SWD, whose insomnia is primarily attributed to circadian disruptions and misalignments due to shift work.

To summarize, based on our study CBT-I should only be considered if a shift worker with insomnia has clinically significant symptoms regardless of their shifts, or if they have comorbid mental disorders or symptoms. Likewise, certain studies show that CBT-I is effective among insomnia patients with comorbid mental disorders and occasionally also improves the comorbid disorder (Taylor and Pruiksma, 2014). In addition, current evidence suggests that preventing mental health problems by CBT-I may be possible (Freeman et al. 2017), which supports using CBT-I also with patients without present mental disorders. In our study, based on the preference of the shift workers themselves, both CBT-I interventions showed superiority over short SH education. However, our results also reveal overall uncertainty regarding the usefulness of g- and sCBT-I interventions among shift workers. This may be because of the sleep-disturbing characteristics of the participants’ work schedules (Flo et al. 2014; Waage et al. 2014), which make improving their sleep through interventions impossible, as only individual factors are targeted. Sleep–wake disturbances can also be decreased through ergonomic shift scheduling by e.g. minimizing the proportion of night shifts (Harma et al. 2018; Sallinen and Kecklund 2010). In addition, OHSs should take individual tolerance to shift work into account (Saksvik et al. 2011). None of our participants had an SWD diagnosis, although half were evaluated as having SWD features. One possible reason for this is that OHS professionals have insufficient knowledge and experience on the evaluation of SWD. Overall, shift workers with insomnia probably have many etiological factors behind their symptoms. Thus, the transdiagnostic sleep treatment approach (Harvey and Buysse 2018) may be the best solution. This approach integrates the elements of CBT-I, interpersonal and social rhythm therapy, and other evidence-based therapies to find an individually fitting and effective treatment for complex and comorbid sleep and circadian rhythm problems. Our study also used such a modified CBT-I approach. In practice, the interventions of our study could be used and tailored based on the individual needs of each shift worker. For example, SWD-type insomnia may improve enough after short SH and circadian adaption education and individual rescheduling of shifts if needed, whereas those with insomnia independent of working hours probably need more intensive CBT-I. In addition, these two perspectives should be combined if SWD and an insomnia disorder coexist.

Certain factors limit the generalizability of our results. The small number of differences between the interventions limits or contests any conclusions. In addition, we had no “no treatment” control group for assessing the overall effects of all the interventions. The comparable results between interventions may be explained by e.g. the intervention effect of the measurements, the effect caused by receiving attention to the sleeping problem, or through natural variation in insomnia-related symptoms. More effective treatment for this special group of insomniacs may have required more experience and expertise from the treatment providers. Our sample size may not have been sufficient to detect differences between the interventions. The recruited sample was slightly smaller than planned based on the power calculations made during the planning stage of the study. In addition, more participants discontinued than in our previous study (Jarnefelt et al. 2012), which decreased the final sample size and probably reduced the power of our results. One reason for discontinuation may have been the participants’ perceived poor suitability of these interventions for improving their sleeping problems. Another possible explanation for the weaker result is that attendance in the group meetings was considerably weaker in this study than in our previous study (1/24 vs. 12/23 of the participants attending all sessions) (Jarnefelt et al. 2012), in which the participants could attend during working hours. Employers should be encouraged to allow their employees to attend treatments such as this during working hours, if necessary. Otherwise, we did not gather systematic information concerning intervention compliance among participants. Neither were the participants blinded for the intervention type. This may have negatively affected response expectations and appraisals, especially among the SH education participants because they had only one intervention session. The small sample size also made it impossible to compare which intervention works best for insomniacs with or without SWD features. In addition, based on the sleep diaries and actigraphy, our participants’ sleep was not severely impaired and this may have affected the degree to which their sleep could have improved (ceiling effect), as also shown in our earlier CBT-I study among both day and shift workers (Jarnefelt et al. 2014). We recommend paying more attention to this probable ceiling effect when recruiting participants for future sleep intervention studies through OHS.

The generalizability of our findings also faces certain other limitations. Airline participants were not randomized into gCBT-I in the current study. Although it was quite a small group, mainly containing cabin attendants (12% of the sample), it may have affected the sCBT-I and SH education results because of the typically very irregular and long working hours of this group, which possibly impair their sleep more than the working times in other professional fields. This shows at least a practical limitation to implementing gCBT-I in certain occupations. In addition, participants recruited by OHS and FIOH were slightly different in terms of shift work duration and professional field. The partly different recruitment processes and expertise in assessment and intervention methods in OHS and FIOH may have affected the sample and results. Participants recruited by FIOH were possibly more suitable for the study because they were preincluded through a short electronic questionnaire. In addition, FIOH had more previous expertise in sleep medicine and intervention methods and this may have positively affected the results. However, the participants were distributed unequally in the interventions in terms of this recruitment factor, which made the analysis of these effects unreliable. The shift work inclusion criteria for our study was also quite loose (only a 10% minimum of shifts had to be other than day shifts), making the generalizability of the results more uncertain because shift workers’ working times and the circadian disruption and misalignment related to these vary greatly. Although most of our participants clearly exceeded the minimum criteria, future studies should consider stricter, multi-dimensional criteria (Harma et al. 2015; Vanttola et al. 2019). We should also note that the overall sample was quite small and three out of four participants were women. However, insomnia is generally more common in women (Morin et al. 2015), and they are typically over-represented (approximately 60% of participants) in clinical non-pharmacological insomnia trials (Morin et al. 1999). In our current study, over half of our participants worked in professional fields consisting predominantly of female employees (e.g. nurses and cabin attendants). Future studies should test insomnia treatments in shift work fields consisting of more male employees.

This clinical RCT trial was implemented in different OHS contexts, showing that OHS could be a good route for delivering such treatments. We took SWD into account in our analyses as a potentially important background factor for insomnia among this group of people, and this is the strength of our study. There are still only a few studies on CBT-I or other non-pharmacological treatments of insomnia among shift workers, which makes it difficult to frame practical treatment guidelines in this case. Future insomnia treatment studies should include potential SWD already during the screening and randomization of the participants to determine the best practices for shift workers with insomnia.

## References

[CR1] Aalto AM, Aro AR, Teperi J (1999) RAND-36 as a measure of health-related quality of life. Realibility, construct validity and reference values in Finnish general population. Helsinki: Stakes. https://www.julkari.fi/bitstream/handle/10024/76006/Tu101.pdf?s

[CR2] American Academy of Sleep Medicine (2014). international classification of sleep disorders; diagnostic and coding manual.

[CR3] Baglioni C, Battagliese G, Feige B, Spiegelhalder K, Nissen C, Voderholzer U, Riemann D (2012). Insomnia as a predictor of depression: a meta-analytic evaluation of longitudinal epidemiological studies. J Affect Disord.

[CR4] Bastien CH, Vallieres A, Morin CM (2001). Validation of the Insomnia Severity Index as an outcome measure for insomnia research. Sleep Med.

[CR5] Beck AT, Steer RA, Garbin MG (1988). Psychometric properties of the Beck Depression Inventory: twenty-five years of evaluation. Clin Psychol Rev.

[CR6] Buysse DJ, Ancoli-Israel S, Edinger JD, Lichstein KL, Morin CM (2006). Recommendations for a standard research assessment of insomnia. Sleep.

[CR7] Carney CE, Buysse DJ, Ancoli-Israel S, Edinger JD, Krystal AD, Lichstein KL, Morin CM (2012). The consensus sleep diary: standardizing prospective sleep self-monitoring. Sleep.

[CR8] Cheng SK, Dizon J (2012). Computerised cognitive behavioural therapy for insomnia: a systematic review and meta-analysis. Psychother Psychosom.

[CR9] Drake CL, Roehrs T, Richardson G, Walsh JK, Roth T (2004). Shift work sleep disorder: prevalence and consequences beyond that of symptomatic day workers. Sleep.

[CR10] Eurofound (2017). Sixth European working conditions survey overview report.

[CR11] Flo E, Pallesen S, Moen BE, Waage S, Bjorvatn B (2014). Short rest periods between work shifts predict sleep and health problems in nurses at 1-year follow-up. Occup Environ Med.

[CR12] Fortier-Brochu E, Beaulieu-Bonneau S, Ivers H, Morin CM (2012). Insomnia and daytime cognitive performance: a meta-analysis. Sleep Med Rev.

[CR13] Freeman D, Sheaves B, Goodwin GM, Yu LM, Nickless A, Harrison PJ, Espie CA (2017). The effects of improving sleep on mental health (OASIS): a randomised controlled trial with mediation analysis. Lancet Psychiatry.

[CR14] Harma M, Ropponen A, Hakola T, Koskinen A, Vanttola P, Puttonen S, Kivimaki M (2015). Developing register-based measures for assessment of working time patterns for epidemiologic studies. Scand J Work Environ Health.

[CR15] Harma M, Karhula K, Ropponen A, Puttonen S, Koskinen A, Ojajarvi A, Kivimaki M (2018). Association of changes in work shifts and shift intensity with change in fatigue and disturbed sleep: a within-subject study. Scand J Work Environ Health.

[CR16] Harvey AG, Buysse DJ (2018). Treating sleep problems a transdiagnostic approach.

[CR17] Hays RD, Sherbourne CD, Mazel RM (1993). The RAND 36-Item Health Survey 1.0. Health Econ.

[CR18] Irish LA, Kline CE, Gunn HE, Buysse DJ, Hall MH (2015). The role of sleep hygiene in promoting public health: a review of empirical evidence. Sleep Med Rev.

[CR19] Jacobs GD, Pace-Schott EF, Stickgold R, Otto MW (2004). Cognitive behavior therapy and pharmacotherapy for insomnia: a randomized controlled trial and direct comparison. Arch Intern Med.

[CR20] Jarnefelt H, Lagerstedt R, Kajaste S, Sallinen M, Savolainen A, Hublin C (2012). Cognitive behavioral therapy for shift workers with chronic insomnia. Sleep Med.

[CR21] Jarnefelt H, Sallinen M, Luukkonen R, Kajaste S, Savolainen A, Hublin C (2014). Cognitive behavioral therapy for chronic insomnia in occupational health services: analyses of outcomes up to 24 months post-treatment. Behav Res Ther.

[CR22] Jarvinen O, Saarinen T, Julkunen J, Huhtala H, Tarkka MR (2003). Changes in health-related quality of life and functional capacity following coronary artery bypass graft surgery. Eur J Cardiothorac Surg.

[CR23] Kecklund G, Axelsson J (2016). Health consequences of shift work and insufficient sleep. BMJ.

[CR24] Kerkhof GA (2018). Shift work and sleep disorder comorbidity tend to go hand in hand. Chronobiol Int.

[CR25] Kinnunen U, Feldt T (2013). Job characteristics, recovery experiences and occupational well-being: testing cross-lagged relationships across 1 year. Stress Health.

[CR26] Kronholm E, Partonen T, Harma M, Hublin C, Lallukka T, Peltonen M, Laatikainen T (2016). Prevalence of insomnia-related symptoms continues to increase in the Finnish working-age population. J Sleep Res.

[CR27] Kucharczyk ER, Morgan K, Hall AP (2012). The occupational impact of sleep quality and insomnia symptoms. Sleep Med Rev.

[CR28] Lappalainen K, Aminoff M, Hakulinen H, Hirvonen M, Räsänen K, Sauni R, Stengård J (2016). Occupational healthcare in Finland 2015.

[CR29] Meyer TJ, Miller ML, Metzger RL, Borkovec TD (1990). Development and validation of the Penn State Worry Questionnaire. Behav Res Ther.

[CR30] Morin CM, Hauri PJ, Espie CA, Spielman AJ, Buysse DJ, Bootzin RR (1999). Nonpharmacologic treatment of chronic insomnia. An American academy of sleep medicine review. Sleep.

[CR31] Morin CM, Vallieres A, Ivers H (2007). Dysfunctional beliefs and attitudes about sleep (DBAS): validation of a brief version (DBAS-16). Sleep.

[CR32] Morin CM, Belleville G, Belanger L, Ivers H (2011). The Insomnia Severity Index: psychometric indicators to detect insomnia cases and evaluate treatment response. Sleep.

[CR33] Morin CM, Drake CL, Harvey AG, Krystal AD, Manber R, Riemann D, Spiegelhalder K (2015). Insomnia disorder. Nat Rev Dis Primers.

[CR34] Okajima I, Komada Y, Inoue Y (2011). A meta-analysis on the treatment effectiveness of cognitive behavioral therapy for primary insomnia. Sleep Biol Rhythms.

[CR35] Partinen M, Gislason T (1995). Basic Nordic Sleep Questionnaire (BNSQ): a quantitated measure of subjective sleep complaints. J Sleep Res.

[CR36] Partinen M, Hublin C, Kryger M, Roth T, Dement WC (2011). Epidemiology of sleep disorders. Principles and practice of sleep medicine.

[CR37] Peter L, Reindl R, Zauter S, Hillemacher T, Richter K (2019). Effectiveness of an online CBT-I intervention and a face-to-face treatment for shift work sleep disorder: a comparison of sleep diary data. Int J Environ Res Public Health.

[CR38] Riemann D, Baglioni C, Bassetti C, Bjorvatn B, Dolenc Groselj L, Ellis JG, Spiegelhalder K (2017). European guideline for the diagnosis and treatment of insomnia. J Sleep Res.

[CR39] Saksvik IB, Bjorvatn B, Hetland H, Sandal GM, Pallesen S (2011). Individual differences in tolerance to shift work–a systematic review. Sleep Med Rev.

[CR40] Sallinen M, Kecklund G (2010). Shift work, sleep, and sleepiness differences between shift schedules and systems. Scand J Work Environ Health.

[CR41] Schiller H, Soderstrom M, Lekander M, Rajaleid K, Kecklund G (2018). A randomized controlled intervention of workplace-based group cognitive behavioral therapy for insomnia. Int Arch Occup Environ Health.

[CR42] Seligman MEP (1995). The effectiveness of psychotherapy: the Consumer Reports Study. Am Psychol.

[CR43] Shirom A, Melamed S (2006). A comparison of the construct validity of two burnout measures in two groups of professionals. Int J Stress Manag.

[CR44] Sivertsen B, Lallukka T, Salo P, Pallesen S, Hysing M, Krokstad S, Simon O (2014). Insomnia as a risk factor for ill health: results from the large population-based prospective HUNT Study in Norway. J Sleep Res.

[CR45] Stepanski EJ, Wyatt JK (2003). Use of sleep hygiene in the treatment of insomnia. Sleep Med Rev.

[CR46] Taylor DJ, Pruiksma KE (2014). Cognitive and behavioural therapy for insomnia (CBT-I) in psychiatric populations: a systematic review. Int Rev Psychiatry.

[CR47] Vanttola P, Harma M, Viitasalo K, Hublin C, Virkkala J, Sallinen M, Puttonen S (2018). Sleep and alertness in shift work disorder: findings of a field study. Int Arch Occup Environ Health.

[CR48] Vanttola P, Puttonen S, Karhula K, Oksanen T, Harma M (2019). Prevalence of shift work disorder among hospital personnel: a cross-sectional study using objective working hour data. J Sleep Res.

[CR49] Vgontzas AN, Fernandez-Mendoza J, Liao D, Bixler EO (2013). Insomnia with objective short sleep duration: the most biologically severe phenotype of the disorder. Sleep Med Rev.

[CR500] World Health Organization (1995). International statistical classification of diseases and related health problems (ICD-10), 10 th revision.

[CR50] Waage S, Pallesen S, Moen BE, Mageroy N, Flo E, Di Milia L, Bjorvatn B (2014). Predictors of shift work disorder among nurses: a longitudinal study. Sleep Med.

[CR51] West AC, Bechtold DA (2015). The cost of circadian desynchrony: evidence, insights and open questions. BioEssays.

[CR52] Wickwire EM, Geiger-Brown J, Scharf SM, Drake CL (2017). Shift work and shift work sleep disorder: clinical and organizational perspectives. Chest.

[CR53] Wright KP, Bogan RK, Wyatt JK (2013). Shift work and the assessment and management of shift work disorder (SWD). Sleep Med Rev.

