# Correction to: Cognitive behavioural therapy interventions for insomnia among shift workers: RCT in an occupational health setting

**DOI:** 10.1007/s00420-020-01527-4

**Published:** 2020-03-03

**Authors:** Heli Järnefelt, Mikko Härmä, Mikael Sallinen, Jussi Virkkala, Teemu Paajanen, Kari-Pekka Martimo, Christer Hublin

**Affiliations:** 1 Finnish Institute of Occupational Health (FIOH), Topeliuksenkatu 41 b, 00250 Helsinki, Finland; 2 Department of Psychology and Logopedics, University of Helsinki, Helsinki, Finland; 3 Department of Psychology, University of Jyväskylä, Jyväskylä, Finland; 4 Department of Clinical Neurophysiology, University of Helsinki and Helsinki University Hospital, Helsinki, Finland

## Correction to: International Archives of Occupational and Environmental Health 10.1007/s00420-019-01504-6

In the original publication of the article, the first name and last name of the authors were interchanged. The correct sequence of the author’s name should read as given below:

Heli Järnefelt, Mikko Härmä, Mikael Sallinen, Jussi Virkkala, Teemu Paajanen, Kari-Pekka Martimo, Christer Hublin

The original article has been corrected.

